# Endotoxemia contributes to CD27+ memory B-cell apoptosis via enhanced sensitivity to Fas ligation in patients with Cirrhosis

**DOI:** 10.1038/srep36862

**Published:** 2016-11-18

**Authors:** Li-Yuan Chang, Yonghai Li, David E. Kaplan

**Affiliations:** 1 Medicine and Research Services, Corporal Michael J. Crescenz VA Medical Center, 3900 Woodland Avenue, Philadelphia, PA 19104, USA; 2 Division of Gastroenterology, Department of Medicine, University of Pennsylvania, 9th floor BRB, 421 Curie Blvd, Philadelphia, PA 19104, USA.

## Abstract

Peripheral CD27^+^ memory B-cells become quantitatively reduced and dysfunctional in patients with cirrhosis through poorly characterized mechanisms. We hypothesized that the disappearance of CD27^+^ memory B-cells results from enhanced sensitivity to apoptosis caused by exposure to gut microbial translocation products. Using isolated naïve and memory B-cells from patients with cirrhosis and age-matched controls, *ex vivo* and activation-induced sensitivity to Fas-mediated apoptosis was assessed under relevant experimental conditions. We observed differential expression of CD95(Fas) in CD27^+^ B-cells from cirrhotic patients that was inversely correlated with peripheral CD27^+^ B-cell frequency. While memory B-cells from cirrhotic patients were resistant to Fas-mediated apoptosis *ex vivo*, Toll-like receptor 4(TLR4)-ligation restored Fas-sensitivity. Sensitivity to Fas-mediated apoptosis could be transferred to healthy donor memory B-cells by co-culturing these cells with plasma from cirrhotic patients, a sensitivity partially mediated by Fas and TLR4 signaling, and partially rescued via B-cell receptor crosslinking. We conclude that peripheral CD27^+^ memory B-cells in cirrhosis exhibit increased sensitivity to Fas-induced apoptosis in an activation-dependent manner to which endotoxin contributes, associated with reduced frequency of circulating memory B-cells. Destruction of this critical cell subset may contribute to the cirrhotic immunodeficiency state and heightened risk of systemic infections in advanced liver disease.

Liver cirrhosis predisposes patients to terminal bacterial infections^1^,^2^ previously attributed predominantly to defects of innate immunity^3^,^4^. However, humoral immunity has long been known to be dysfunctional in cirrhosis by unclear mechanisms^5^,^6^,^7^. We have previously shown that profound quantitative and qualitative defects in the memory B-cell compartment exist in cirrhosis^8^. Specifically, we observed that peripheral CD27^+^ memory B-cell frequency amongst CD19^+^ B-cells is approximately 10% in individuals with cirrhosis (either due to chronic hepatitis C (HCV) or metabolic/alcoholic liver disease) compared with 30% in healthy individuals or those with non-cirrhotic HCV infection. The reduction in circulating peripheral B-cells strongly correlated with portal hypertension (using platelet count as a surrogate marker) and hepatic synthetic dysfunction. CD27, a TNF receptor family receptor that is expressed by antigen-experience memory B-cells, interacts with CD70 on T-cells to promote T-cell survival and proliferation while the reciprocal ligation of CD27 by CD70 promotes immunoglobulin production, plasma cell differentiation and survival (reviewed in ref. 9). In our previous work, reduction of CD27+ B-cell frequency in cirrhosis was strongly associated with reduced B-cell cytokine production as well as impaired allostimulation-induced CD4^+^ T-cell proliferation. We suspect that cirrhosis-related CD27+ memory B-cell loss could therefore be a critical contributor to the functional immunodeficiency present in patients with cirrhosis. The dominant mechanism leading to the reduction of circulating memory B-cells remains unknown.

The fate of peripheral memory B-cells in cirrhosis could be determined by altered compartmentalization, differentiation, or destruction. There is no specific evidence for enhanced trafficking or retention of memory B-cells in the liver, secondary lymphoid tissue, or spleen in cirrhosis, but an enrichment of CXCR3^+^ or CXCR5^+^ CD27^+^ memory B-cells in chronic viral hepatitis has been suggested immunohistochemically^10^,^11^. Enhanced differentiation of memory B-cells into plasmablasts in chronic hepatitis has also been suggested as a cause of memory B-cell turnover^12^,^13^. Our group identified a small increase in CD27^−^CD21^−^ hypoproliferative tissue-like memory B-cells in patients with viral hepatitis-related cirrhosis that could not numerically account for the disappearance of CD27^+^ B-cells nor account for the similar reduction observed in non-viral-related cirrhosis^14^. The role of apoptosis of B-cells in cirrhosis has not previously specifically been reported, but results of investigations in chronic viral hepatitis have yielded conflicting results. Some groups have reported an increased sensitivity to apoptosis of naïve B-cells in HCV-infected patients with mixed cryoglobulinemia, but not in non-cryoglobulinemic subjects^15^, a finding which may not be generalizable to non-cryoglobulinemic patients nor memory B-cell subsets^16^,^17^,^18^.

The objective of this study was to characterize the role of apoptosis in the disappearance of peripheral CD27^+^ memory B-cells. We found that while CD27^+^ memory B-cells from cirrhotic patients displayed increased expression of the death receptor CD95 (Fas), these cells paradoxically were resistant to Fas-mediated apoptosis *ex vivo*. Upon CD40- or TLR4-mediated activation, memory B-cells from cirrhotic patients regained sensitivity to Fas-mediated apoptosis much more significantly than naïve B-cells or memory B-cells from healthy subjects. Potential exposure of circulating B-cells to soluble Fas Ligand (sFasL) and membrane-associated Fas Ligand (mFasL) are increased in cirrhotic patients. Notably, cirrhotic plasma induced apoptosis in healthy donor memory B-cells as well as increased their sensitivity to Fas-mediated apoptosis, phenomena partially mediated by sFasL and lipopolysaccharide (LPS). These studies strongly implicate Fas-mediated apoptosis as a key mechanism of memory B-cell loss in cirrhosis and may contribute to the impairment of innate and adaptive immunity characteristic of end-stage liver disease.

## Patients, Materials and Methods

### Patients

Subjects and controls were recruited from the Gastroenterology Clinic at the Corporal Michael J. Crescenz Veterans Affairs Medical Center (CMCVAMC). The protocol was approved by the CMCVAMC institutional review board and all activities were performed in accordance with The Common Rule (45 CFR part 46) and applicable US Department of Veterans Affairs regulations. Informed consent was obtained from all patients. Due to the volume of blood draw required to obtain adequate lymphocytes for study, decompensated cirrhotic patients were excluded. Viral hepatitis, alcohol abuse, hemochromatosis, and non-alcoholic fatty liver disease/non-alcoholic steatohepatitis diagnoses were obtained from clinical records. Cirrhotic patients were routinely screened by sonography every 6 months to exclude interim development of HCC; data from any cirrhotic subject who developed HCC within 12 months of enrollment were excluded.

### Cell isolation and preparation

100–150 ml of peripheral blood was obtained, from which 100–200 million peripheral blood mononuclear cells (PBMC) were isolated using Ficoll-Histopaque (Sigma, St. Louis MO) density gradient centrifugation. B-cells were purified from 100 × 10^6^ PBMC by negative selection using the MACS B-cell Isolation Kit II (Miltenyi Biotec, Bergisch Gladbach, Germany). CD27^+^ Memory B cells were isolated from purified B cells by positive selection using CD27 MicroBeads (Miltenyi Biotec, Bergisch Gladbach, Germany). Purity of CD27^+^ and CD27^−^ B cells were >90% as determined by flow cytometry. CD27^+^ or CD27^−^ B cells were plated in 96 well plates in RPMI1640 with L-glutamine (Invitrogen) with 10% human AB serum (Sigma Inc., St. Louis, MO), 1.5% HEPES (Invitrogen) and 1% penicillin/streptomycin (Invitrogen).

### Antibodies and flow cytometry

All data were acquired on FACSCanto (BD: Becton Dickinson, San Jose, CA) and analyzed using FlowJo (Tree Star Inc., Ashland OR) using cutoffs based on isotype antibody staining. All antibodies were purchased from Becton Dickinson (BD: Becton Dickinson, Franklin Lakes, NJ) except for anti-bcl-2 (eBioscience, San Diego, CA), anti-Bcl-xL (Santa Cruz Biotechnology, Dallas, TX), and a fixable Live/Dead Aqua Staining kit (Invitrogen).

### *In vitro* activation/apoptosis inductinon

1 × 10^5^ CD27^+^ or CD27^−^ B-cells were incubated with agonistic anti-CD40 mAb (1 μg/ml, CP-870,893; kindly provided by Pfizer, New London, CT), the dsRNA complex polyinosinic:polycytidylic acid (10 μg/ml, poly(I:C); Sigma), lipopolysaccharide (10 μg/ml, LPS; Sigma), resiquimod (10 μg/ml, R848; Sigma) or CpG oligodeoxynucleotide (ODN) 2006 (1 μg/ml, InvivoGen, San Diego, CA). After 48 hours, cells were washed in complete medium and were then cultured in complete medium containing agonistic anti-CD95 mAb (2 μg/ml, CH11; MBL International, Woburn, MA) or human rTRAIL (1 μg/ml; R&D Systems, Minneapolis, MN) for an additional 18 hours. In confirmatory experiments, an alternative anti-CD95 mAb (SM1/1, eBioscience, San Diego, CA) or a sFasL (R&D Systems, Minneapolis, MN) incubated with anti-His Tag (R&D Systems, Minneapolis, MN) to allow cross-linking of Fas receptors. Cells were then washed in PBS and resuspended in Annexin-V binding buffer with Annexin-V-FITC and propidium iodide (PI; BioLegend, San Diego, CA). The apoptosis induced by adding agonistic anti-CD95 mAb or rTRAIL was defined as the change in % Annexin-V^+^ and calculated by subtracting the value for percentage of Annexin-V-positive cells in culture medium alone (background apoptosis) from the value for percentage of apoptosis in a replicate culture containing agonist anti-Fas mAb or rTRAIL.

### Plasma co-culture

1 × 10^5^ CD27^+^ B-cells from healthy donors were cultured in 50% complete medium supplemented with 50% plasma from either CIR or HD with or without 2 μg/ml agonist anti-Fas mAb (CH11; MBL International, Woburn, MA). In some experiments, the TLR4-antagonist *Rhodobacter sphaeroides* LPS (10 μg/ml, LPS/RS; InvivoGen, San Diego, CA), blocking anti-BAFF mAb(20 μg/ml, 148725; R&D Systems, Minneapolis, MN), blocking anti-Fas mAb (10 μg/ml, SM1/23; eBioscience, San Diego, CA), blocking anti-CD40L mAb (10 μg/ml, MK13A4; Enzo Life Sciences, Farmingdale, NY). In some experiments, HD CD27+ B-cells were preincubated for 30 minutes with agonistic IgG/A/M (20 μg/ml, Jackson Immunolabs, Kennett Square PA) or anti-Fc receptor mAb (BioLegend, San Diego, CA). For neutralizing circulating Immunoglobulin (Ig) in CIR plasma, circulating Ig were removed by protein A/G (Spherotech, Lake Forest, IL) before co-cultured with CD27^+^ B-cells. After 18 hours, cells were then washed in PBS and resuspended in Annexin-V binding buffer with Annexin-V-FITC and PI (BioLegend, San Diego, CA).

### Exosome isolation

For selected co-culture experiments, exosomes were isolated from HD and CIR plasma utilizing Total Exosome Isolation Reagent (Invitrogen, San Diego CA) per manufacturer’s instructions.

### Enzyme-linked immunosorbent assay

sFasL and sCD40L quantities in plasma were tested (freshly frozen and stored at −80c) using ELISA kits (R&D Systems, Minneapolis, MN) according to the manufacturer’s instructions. Plasma LPS was measured using the Limulus Amoebocyte Assay (Pierce Biotechnology, Rockford IL) according to manufacturer’s instructions.

### Statistical Analysis

Median values for clinical and immunologic parameters were compared using Wilcoxon signed-rank test, the nonparametric Kruskal-Wallis, or Wilcoxon Rank Sum test. All Statistical Analysis were performed using JMP Pro 12 (SAS Institute Inc, Cary NC). P-values of < 0.05 were considered significant.

## Results

### Patient Characteristics

The study cohort comprised of 45 subjects, 26 with liver cirrhosis and 19 healthy donors (Table 1, Supplemental Table 1). The median age of cirrhotic patients was slightly higher than the healthy donors; however only 2 cirrhotic patients and 2 healthy donors were under the age 50 years old. Cirrhotic patients were compensated with relatively normal serum albumin, serum bilirubin and INR values. As expected due to portal hypertension, median platelet counts were lower in the cirrhotic group (146 versus 225 × 10^3^/μl, p < 0.0001).

### Reduced frequency of circulating CD27^+^ memory B-cells is associated with increased expression of Fas (CD95)

**Figure 1:**
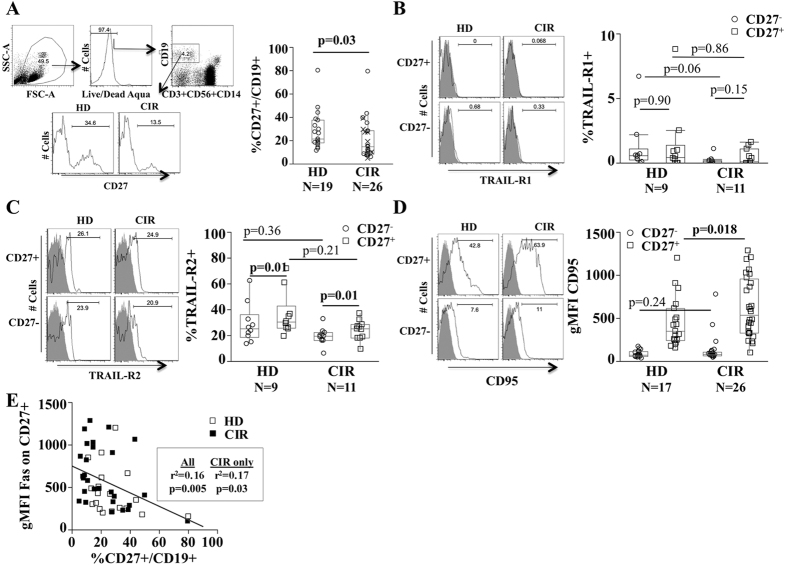
Increased expression of pro-apoptotic receptor Fas (CD95) on CD27^+^ B-cells in cirrhosis. (**A**) Representative FACS analyses and quantification of CD27 expression on CD19^+^ B-cells in healthy donors (HD) and cirrhotic patients (CIR). X marker indicates non-viral cirrhosis. Representative histograms and quantification of TRAIL-R1 (**B**), TRAIL-R2 (**C**) and CD95 (**D**) expression on CD27^+^ B-cells and CD27^−^ B-cells in HD and CIR. Gray histogram is isotype control. Box plots represent median and interquartile range. Circles represent CD27^−^ and boxes CD27^+^ B-cells. (**E**) Spearman correlation of CD27^+^ B-cell frequency and CD95 expression *ex vivo*.

Consistent with our prior observation, the frequency of CD27^+^ memory B-cells was reduced in patients with cirrhosis (CIR) relative to healthy donors (HD) (28.3 ± 3.4 in HD versus 19.5 ± 2.4 in CIR, p = 0.029, Fig. 1A). We evaluated which pro-/anti-apoptotic receptors and/or transcription factors might differ in expression between CIR and HD B-cells. The pro-survival BAFF receptors Transmembrane activator and CAML interactor (TACI, CD267) as well as transcription factors bcl-2 and bcl-xl were expressed more strongly in memory CD27^+^ relative to naïve CD27^−^ B-cells but did not differ between HD and CIR (data not shown). By contrast, the B cell-activating factor belonging to the TNF family (BAFF)-receptor (CD268) was more highly expressed on naïve B-cells. TNF-related apoptosis-inducing ligand (TRAIL)-R1 expression was minimal on both HD and CIR B-cells (Fig. 1B) while TRAIL-R2 was more frequently present on memory B-cells at similar frequencies in both CIR and HD (Fig. 1C) and similar geometric mean fluorescence intensity (gMFI) (data not shown). The pro-apoptotic CD95 (Fas) was nearly exclusively expressed on CD27^+^ memory B-cells and we noted a small but statistically significant increase of expression in CIR CD27^+^ B-cells (gMFI 378.4 ± 47.3 in HD versus gMFI 615.8 ± 65.1 in CIR, p = 0.018, Fig. 1D). Furthermore, CD95 expression was moderately inversely associated with CD27^+^ B-cell frequency (R^2^ = 0.17, p = 0.04, Fig. 1E). The expression of CD95 on memory B-cells did not vary with patient age (among healthy donors or cirrhotic patients) nor with the etiology of cirrhosis (viral versus non-viral) (Supplemental Fig. 1). These data suggested a possible association of CD95 and the reduction of circulating memory B-cells in cirrhosis.

### CD27^+^ memory B-cells from cirrhotic patients are insensitive to Fas-mediated apoptosis *ex vivo* but regain sensitivity after activation

**Figure 2:**
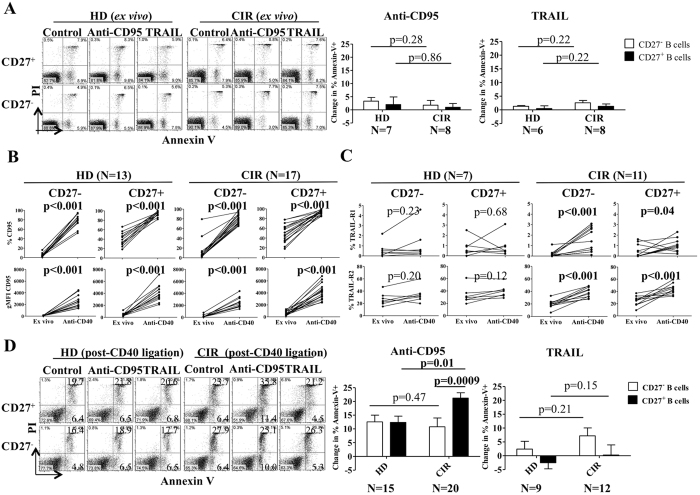
Increased sensitivity to Fas-mediated apoptosis in anti-CD40-activated CD27^+^ B-cells in cirrhosis. (**A**) Purified CD27^+^ and CD27^−^ B-cells from HD and CIR were incubated with agonistic anti-CD95 mAb (CH11) and recombinant TNF-related apoptosis-inducing ligand (rTRAIL) for 18 hours and then labeled with Annexin-V-FITC and propidium iodide (PI). Cumulative data showing the change in % Annexin-V^+^ using by subtracting the value for percentage of Annexin-V-positive cells in culture medium alone from the value for percentage of apoptosis in a replicate culture containing agonistic anti-CD95 mAb or rTRAIL. Error bars reflect standard deviation. All comparisons tested with Wilcoxon test. (**B**) Purified CD27^+^ and CD27^−^ B-cells from HD and CIR were incubated with agonistic anti-CD40 mAb for 48 hours. The data represent the frequency and geometric mean fluorescence (gMFI) of CD95 on CD27^+^ and CD27^−^ B-cells *ex vivo* and after CD40 ligation. All comparisons tested with Wilcoxon test. (**C**) The frequency of TRAIL-R1 and TRAIL-R2 on CD27+ and CD27- B-cells *ex vivo* and after CD40 ligation. (**D**) After CD40 ligation, CD27^+^ and CD27^−^ B-cells were incubated with agonistic anti-CD95 mAb (CH11) and rTRAIL for an additional 18 hours and then labeled with Annexin-V-FITC and propidium iodide (PI). Cumulative data showing the change in % Annexin-V+ using by subtracting the value for percentage of Annexin-V-positive cells in culture medium alone from the value for percentage of apoptosis in a replicate culture containing agonistic anti-CD95 mAb or rTRAIL. Error bars reflect standard deviation. All comparisons tested with Wilcoxon test.

To test the Fas sensitivity of memory B-cells relative to naïve B-cells, purified CD27^+^ and CD27^−^ B-cells from CIR and HD subjects were co-cultured with a Fas-agonizing antibody (CH11, 2 μg/ml) or rhTRAIL (1 μg/ml) for 18 hours after which apoptosis was assessed by Annexin-V/propidium iodide staining. Despite elevated Fas and TRAIL-R2 expression, memory B-cells from HD and CIR were insensitive to Fas- or TRAIL-induced apoptosis *ex vivo* (Fig. 2A). This insensitivity could not be overcome by using alternative Fas agonists or cross-linked soluble Fas Ligand (sFasL) (data not shown). Prior studies have indicated that CD40-, other TNF family receptor-, or LPS- mediated activation can increase murine or neoplastic B-cell sensitivity to apoptosis by upregulating CD95^19^,^20^,^21^. We found that CD40 agonism strongly upregulated CD95 expression on CIR and HD CD27^−^ and CD27^+^ B-cells (Fig. 2B) but upregulated TRAIL-R1 and -R2 expression on CIR B-cells (CD27^−^ and CD27^+^) only (Fig. 2C). Post-activation expression levels of CD95 on memory B-cells did not vary with patient age (among healthy donors or cirrhotic patients) nor the etiology of cirrhosis (viral versus non-viral) (Supplemental Fig. 1). After CD40 activation, naïve (both CIR and HD) and HD memory B-cells exhibited a modest increased sensitivity to Fas-mediated apoptosis compared to *ex vivo* (~2.5% as shown in Fig. 2A to ~10%, ~4-fold); however, CIR CD27^+^ B-cells demonstrated a disproportionate increase in this sensitivity (~2% to ~22%, ~11-fold) (Fig. 2D). In the memory B-cell population, apoptosis sensitivity strongly correlated with Fas levels post-activation by CD40 ligation (R^2^ = 0.33, p = 0.008) but not basal Fas expression levels (Supplemental Fig. 2). By contrast, CD40 activation did not increase TRAIL-mediated apoptosis. After activation, CD27^+^IgM^+^ B-cells from cirrhotic subjects exhibited greater Fas-mediated apoptosis than CD27^+^IgM^−^ B-cells (Supplemental Fig. 3). Furthermore, CD27^+^IgM^+^ healthy donor B-cells had increased propensity to apoptose *ex vivo* when cultured with either HD or CIR plasma possibly explaining our prior observation showing a more prominent reduction of CD27^+^IgM^+^ B-cells in cirrhosis^8^. Thus, while resistant *ex vivo*, CIR CD27^+^ (IgM^+^ to a greater degree than IgM^−^) B-cells became highly sensitive to Fas-mediated but not TRAIL-mediated apoptosis after CD40-mediated activation. This sensitivity was markedly greater than that seen in HD CD27^+^ B-cells despite similar magnitudes of activation-induced Fas upregulation.

### Cirrhosis is associated with increased exposure of B-cells to Fas ligands as well as activating CD40 and TLR agonists

**Figure 3:**
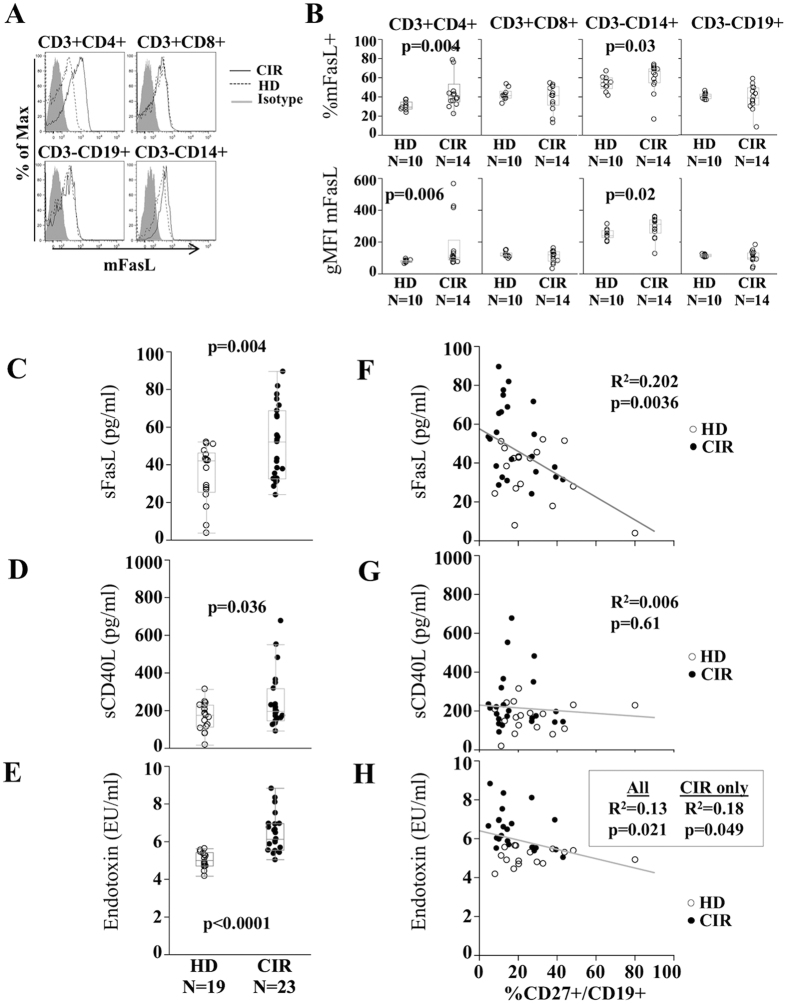
Plasma sFasL is associated with CD27^+^ B-cell frequency in cirrhosis. Representative histograms (**A**) and quantification (**B**) of membrane-associated FasL (mFasL) expression on CD3^+^CD4^+^ cells, CD3^+^CD8^+^ cells, CD3^−^CD19^+^ cells and CD3^−^CD14^+^ cells in HD (dashed) and CIR (solid). Box plots reflect median and interquartile range. P-value by Wilcoxon test. Plasma concentrations by ELISA in HD and CIR of (**C**) soluble FasL (sFasL), (**D**) CD40L (sCD40L) and (**E**) plasma endotoxin (LPS) by Limulus Amaeboctye Assay. Spearman rank correlations of (**F**) plasma sFasL, (**G**) plasma sCD40L, and (**H**) plasma endotoxin and CD27^+^ B-cell frequency in CIR (closed circles) and HD (open circles).

To determine possible differential sources of Fas-ligation for induction of apoptosis in cirrhotic B-cells, we examined which cellular subsets might express membrane-associated Fas Ligand (mFasL) in PBMC. As shown in Fig. 3A,B, CD3+CD4^+^ T-cells and to a lesser extent CD14+ monocytes shows significantly higher expression of mFasL in CIR relative to HD (CD4+mFasL+ median in CIR 40.3% versus 29.1% in HD, p = 0.004). Differences between CIR and HD were partially driven by three individual CIR patients with markedly higher CD3+CD4+ mFasL. Of these three, two had HCV and one had NASH; all 3 had platelet counts <150 K/mm^3^. By Wilcoxon Rank Sum test, there was a trend among cirrhotic patients for platelet counts <150 K/mm^3^ to exhibit higher mFasL (p = 0.09) despite the limited sample size. By contrast, membrane-associated CD40 Ligand (mCD40L) expression on CIR and HD CD3+CD4^+^ T-cells similar, but elevated on CD3+CD8+ T-cells in some CIR patients (Supplemental Fig. 4) again associated with platelet counts <150 K/mm^3^ (data not shown). Plasma levels of sFasL and to a lesser extent sCD40L were significantly greater in CIR than HD (Fig. 3C,D). Of these, only levels of sFasL appeared associated with CD27^+^ memory B-cell frequency (R^2^ = 0.202, p = 0.0036 for sFasL, Fig. 3F,G). We previously showed that soluble CD14 (sCD14) levels were increased in cirrhosis and inversely correlated with memory B-cell frequency^8^. sCD14 interacting with endotoxin has been shown to activate B-cells by binding membrane-associated MD-2^22^. We found that, consistent with previous reports^23^, plasma endotoxin levels were also significantly greater in CIR patients (Fig. 3E). LPS levels also inversely correlated with CD27^+^ memory B-cell frequency (Fig. 3H). Thus, the cirrhotic state is associated with increased levels of both cell-surface and soluble FasL, as well as increased TLR4-interacting ligands sCD14 and LPS, that are associated with reduced circulating memory B-cell frequency.

### Soluble factors from cirrhotic plasma induce sensitivity to Fas-mediated apoptosis in healthy donor B-cells

**Figure 4:**
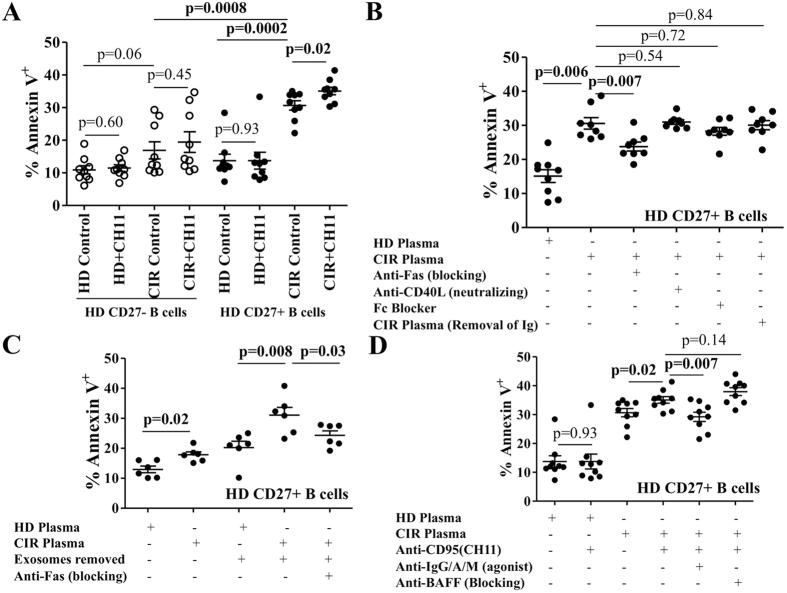
Cirrhotic plasma induces sensitivity to Fas-mediated apoptosis in healthy donor CD27^+^ B-cells. (**A**) Purified CD27^+^ and CD27^−^ B-cells from HD were cultured with HD and CIR plasma in the presence and absence of agonistic anti-CD95 mAb (CH11) for 18 hours and then labeled with Annexin-V-FITC and PI. The data represent the frequency of apoptotic cells (Annexin-V^+^) on CD27^+^ and CD27^−^ B-cells with each plasma sample tested. Error bars reflect standard error of the mean. Statistical comparisons done by Wilcoxon test. (**B**) Impact of blocking anti-Fas mAb, blocking anti-CD40L mAb and two approaches to neutralize circulating Immunoglobulin (Ig) (Fc Blocker and protein A/G-treated CIR plasma) on the expression of Annexin-V on healthy donor CD27^+^ B-cells co-cultured with CIR plasma for 18 hours. (**C**) Impact of removal and re-introduction of exosomes on healthy donor CD27^+^ B-cells co-cultured with HD or CIR plasma with or without inhibition of Fas for 18 hours. Representative data from three separate experiments with different B-cell donors are shown. (**D**) Impact of agonist IgG/A/M (pre-cultured for 30 minutes) and/or blocking BAFF mAb on the expression of Annexin-V on healthy donor CD27^+^ B-cells co-cultured with CIR plasma plus agonistic anti-Fas mAb (CH11) for 18 hours. Representative data from three separate experiments with different B-cell donors are shown. Error bars reflect standard error of the mean. Statistical comparisons done by Wilcoxon test.

We next tested whether or not soluble factors including sFasL and sCD14/LPS present in cirrhotic plasma could alter the sensitivity to apoptosis in healthy donor B-cells. Healthy donor B-cells were cultured in the presence of 50% HD or CIR plasma with or without anti-Fas (CH11) for 18 h and assessed for apoptosis by Annexin V/PI staining. In all experiments, culture of HD B-cells with HD plasma yielded no increase in apoptosis over levels obtained with complete media (data not shown). As shown in Fig. 4A, CIR plasma nonsignificantly increased apoptosis in healthy donor CD27^−^ B-cells and did not confer Fas-sensitivity. In the experiment shown 3 cirrhotic plasma samples, 2 viral and 1 NASH, caused more profound apoptosis; 2 of these had platelet counts <100 K/mm^3^ suggesting that portal hypertension might be associated with increased pro-apoptotic mediators. By contrast, all CIR plasma samples markedly increased basal apoptosis (13.7 ± 1.9% vs. 30.6 ± 1.4%, p = 0.0002), and further sensitized HD CD27^+^ B-cells to Fas-mediated apoptosis (30.6 ± 1.4%vs. 35.8 ± 1.1%, p = 0.02). We suspected that elevated circulating levels of sFasL in cirrhotic plasma could be contributing to basal apoptosis. As shown in Fig. 4B, neutralization of sFasL, but not sCD40L or circulating Ig, partially abrogated basal apoptosis induced by CIR plasma. These findings appear to contradict findings in other systems in which sFasL *in vitro* fails to activate apoptosis due to lack of crosslinking of Fas receptors^24^,^25^,^26^. Removal of exosomes, a putative source of circulating mFasL^27^, increased CD27^+^ B-cell apoptosis suggesting that exosomes do not contribute to B-cell Fas-mediated apoptosis *in vivo* and may actually provide pro-survival signals (Fig. 4C). In the next experiments, we repeated these conditions omitting the Fas agonist with or without blockade of CD95 or neutralization of CD40L with specific antibodies. As opposed to augmenting apoptosis, blocking sFasL signaling partially reduced HD memory B-cell apoptosis (30.5 ± 1.6% vs. 23.7 ± 1.3%, p = 0.007) whereas neutralization of sCD40L had no impact. Since B cell-activating factor belonging to the TNF family (BAFF)^28^ and B-cell receptor (BCR)-crosslinking^28^,^29^ have been previously shown to potentiate or rescue, respectively, Fas-mediated apoptosis in activated CD27^+^ B-cells, we repeated these experiments either by neutralizing BAFF or by crosslinking BCR with anti-IgG/A/M (done prior to exposure to plasma to prevent neutralization of the agonist antibody) (Fig. 4D) and found that BCR crosslinking but not BAFF partially counteracted the Fas-mediated increase of apoptosis. BCR-crosslinking alone contrastingly increased CIR plasma-induced apoptosis (data not shown). Thus, B-cells in cirrhotic patients are exposed to increased levels of both mFasL on CD4^+^ T-cells and sFasL in plasma. Sensitivity to apoptosis can be transferred to healthy donor B-cells by cirrhotic plasma; this sensitivity is at least partially mediated by sFasL (but not sCD40L, exosomes or immune complexes) and can be partially rescued with BCR crosslinking.

### Toll-Like Receptor (TLR)-activation modulates Fas-sensitivity in cirrhotic memory B-cells

**Figure 5:**
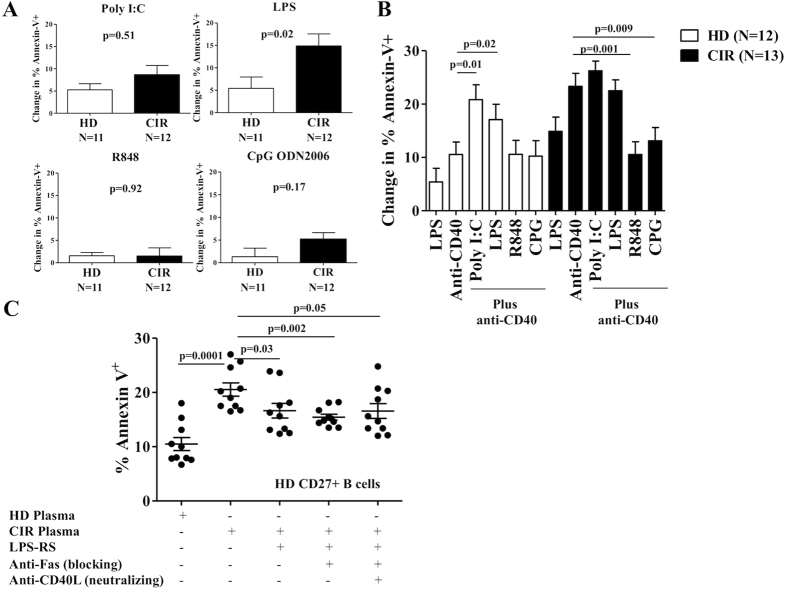
TLR-activation modulates Fas-sensitivity in cirrhotic CD27^+^ B-cells. (**A**) CD27+ B-cells from HD and CIR were incubated with poly(I:C), LPS, R848 and CpG ODN2006 for 48 hours. After TLR ligation, CD27+ B-cells were incubated with agonistic anti-CD95 mAb (CH11) for an additional 18 hours and then labeled with Annexin-V-FITC and PI. Cumulative data showing the change in % Annexin-V^+^ using by subtracting the value for percentage of Annexin-V-positive cells in culture medium alone from the value for percentage of apoptosis in a replicate culture containing agonistic anti-CD95 mAb. (**B**) CD27^+^ B-cells from HD and CIR were incubated with anti-CD40 plus poly(I:C), LPS, R848 and CpG ODN2006 for 48 hours. After CD40/TLR ligation, CD27^+^ B-cells were incubated with agonistic anti-Fas mAb (CH11) for an additional 18 hours and then labeled with Annexin-V-FITC and PI. Cumulative data showing the change in % Annexin-V^+^ using by subtracting the value for percentage of Annexin-V-positive cells in culture medium alone from the value for percentage of apoptosis in a replicate culture containing agonistic anti-CD95 mAb. (**C**) Impact of blocking anti-Fas mAb, blocking anti-CD40L mAb and TLR4 antagonism (inhibitory Lipopolysaccharide Rhodobacter sphaeroides, LPS-RS) on the expression of Annexin-V on healthy donor CD27^+^ B-cells co-cultured with CIR plasma for 18 hours. Representative data from three separate experiments with different B-cell donors are shown. Error bars reflect standard error of the mean. Statistical comparisons done by Wilcoxon test.

Activation of memory B-cells appears critical to Fas-mediated apoptosis, but sCD40L did not appear sufficient for that activation *in vivo* or *ex vivo*. We previously demonstrated that TLR agonists present in cirrhotic plasma activate memory B-cells^8^. We therefore tested whether or not ligation of TLR3, TLR4, TLR7 or TLR9 could increase Fas-mediated B-cell apoptosis at 48 h differentially in CIR and HD. We found that only LPS (endotoxin, TLR4 agonist) and not poly I:C (TLR3 agonist), R848 (TLR7 agonist) or CpG ODN 2006 (TLR9 agonist) when used in isolation significantly increased Fas-mediated B-cell apoptosis in CIR relative to HD (Fig. 5A). Whereas in healthy donor B-cells combining LPS or poly I:C with CD40 rendered these cells sensitive to apoptosis (Fig. 5B, white bars), in cirrhotic CD27^+^ B-cells there was no additive effect of LPS or poly I:C with CD40 activation (Fig. 5B, black bars). By contrast, both TLR7 and TLR9 co-stimulation abrogated CD40L-mediated Fas-sensitivity in CIR CD27^+^ B-cells suggesting a pro-survival effect of these TLRs. Similar to neutralization of sFasL, neutralizing LPS in cirrhotic plasma partially reduced the pro-apoptotic effect of cirrhotic plasma on HD CD27^+^ memory B-cells (Fig. 5C). Interestingly, combining sFasL and LPS neutralization did not fully abrogate cirrhotic plasma-induced Fas-sensitivity, nor did Fc receptor blocking or neutralizing circulating Ig by protein A/G as shown before. Taken together, these data indicate that sensitization to Fas-mediated apoptosis in cirrhosis is partially mediated by LPS *in vivo*. The presence or absence of still unrecognized soluble factors likely also contributes to the Fas-mediated apoptosis observed in cirrhotic patient memory B-cells.

## Discussion

Bacteremia and sepsis are the predominant causes of death in patients with advanced cirrhosis^1^,^2^. Peripheral memory B-cell frequency is markedly decreased in cirrhosis^8^,^30^ and remaining memory B-cells are defective in antigen-presentation capacity^8^. The reduction in circulating peripheral B-cells we previously observed strongly correlated with portal hypertension (using platelet count as a surrogate marker) and hepatic synthetic dysfunction. Loss of CD27^+^ B-cell was strongly associated with reduced cytokine production (such as Tumor-Necrosis Factor β), reduced expression of T-cell costimulation ligands, and impaired allostimulation-induced CD4^+^ T-cells proliferation. Dysregulation of B-cell-T-cell interactions could impair protective humoral and cellular adaptive immunity leading to the susceptibility of cirrhotic patients to lethal bacterial infections. Thus, understanding the mechanisms driving peripheral memory B-cell deficiency in cirrhosis is critical, but to date few data exist. We demonstrate here that peripheral memory B-cells from patients with cirrhosis irrespective of viral or non-viral etiology have profoundly increased sensitivity to activation-induced Fas-mediated, but not TRAIL-mediated, apoptosis, and that soluble factors present in the circulation can transfer this sensitivity to healthy donor memory B-cells.

While not well characterized in cirrhosis, underlying viral hepatitis has been variably associated with altered B-cell apoptosis^16^,^17^,^18^, anergy^13^,^14^,^16^, differentiation^12^, and compartmentalization^10^,^31^. We previously identified a small increase of CD27^−^CD21^−^ hypoproliferative B-cells associated with viral-induced but not metabolic cirrhosis^14^, the magnitude of which could not explain the disappearance of peripheral memory B-cells. Notably, in our prior work, memory B-cell disappearance did not occur in age-matched non-cirrhotic HCV-infected patients but did occur in non-HCV cirrhotics^8^ suggesting memory B-cell apoptosis is specific to the cirrhotic state independent of etiology, an evidence base we increase with this study, with the caveat that causation is difficult to confirm in the human model. Cirrhotic memory B-cells express significantly higher levels of Fas than healthy donor memory B-cells. While *ex vivo* cirrhotic B-cells are resistant to Fas-mediated apoptosis, marked sensitivity could be restored via activation through CD40 ligation or TLR4 agonism. Strikingly, exposing healthy donor memory B-cells to cirrhotic plasma not only strongly increased spontaneous memory B-cell apoptosis but also enhanced sensitization to Fas-mediated apoptosis. This sensitization could be partially abrogated by neutralizing Fas/FasL, blocking LPS-TLR4 interactions, or crosslinking BCR but appears to partially be mediated by the presence or absence of yet undetermined soluble factors. Notably these soluble factors are likely not free Ig, immune complexes or exosomes as pre-clearing plasma of these factors and/or blocking Fc receptors had no impact on B-cell apoptosis.

Due to the chronic nature of the cirrhotic state, it is unclear if the desensitization to Fas-mediated apoptosis we observed *ex vivo* represents the pre-morbid state of memory B-cells *in vivo* or the result of survival of memory B-cells least sensitive to apoptosis over time. That cirrhotic B-cells regain sensitivity to Fas after activation mimics the phenotype of CD27^+^ memory B-cells of certain B-cell malignancies. Basal insensitivity to, followed by CD40-induced enhancement of, Fas-mediated apoptosis *ex vivo* and *in vitro* has been demonstrated in Epstein Barr Virus (EBV)-transformed and neoplastic B-cells^32^,^33^. Fas-resistant lymphoma cell lines frequently exhibit CD40-mediated reversible impairment of the cleavage of caspase-8 due to impairment of death-inducing signal complex (DISC) activity^21^,^34^. In germinal centers, membrane-associated CD40 ligand (mCD40L)-expression by Th1 CD4^+^ T-cells upregulates B-cell Fas expression and renders them sensitive to Fas-mediated cytolysis^35^, a homeostatic process that putatively deletes autoreactive B-cells. We found that mCD40L expression was not significantly increased in peripheral CD4^+^ T-cells in cirrhosis but that plasma sCD40L levels were modestly elevated. However, neutralizing sCD40L had no impact on the pro-apoptotic effects of cirrhotic plasma on healthy donor B-cells raising questions about the role of sCD40L in inducing Fas-sensitivity in cirrhotic B-cells *in vivo*. The insensitivity of post-activation cirrhotic B-cells to TRAIL-mediated apoptosis despite increased sensitivity to Fas-mediated apoptosis requires additional investigation; we could find no other systems in which this observation occurred in the relevant literature.

Chronic antigen exposure, present in cirrhosis due to increased gut microbial translocation appears critical to generating DISC dysfunction in other systems via B-cell receptor (BCR) crosslinking. In immature B-cell lines that have not been exposed to antigen, BCR-crosslinking leads to Fas-Associated protein with Death Domain (FADD)-independent apoptosis^36^,^37^. Perhaps related to this phenomenon, we observed increased apoptosis in healthy donor CD27^+^ B-cells cultured with cirrhotic plasma with anti-IgG/A/M in the absence of Fas ligation. By contrast, in most lymphoma cell lines and settings mimicking chronic antigen exposure, BCR-crosslinking counteracts CD40L sensitization to Fas-mediated apoptosis without abrogating the upregulation of Fas expression^35^, an effect we observed when HD CD27^+^ B-cells exposed to CIR plasma and Fas ligation. At least two independent pathways appear to mediate the effect of BCR-crosslinking: 1) inhibition of Fas-mediated caspase-8 activation possible via the induction of cFLIP_long_^38^,^39^ and 2) prevention of Fas-mediated downregulation of bcl-2/bcl-xl^20^,^35^,^38^. Similar to lymphoma, cirrhosis is a chronic antigen-dependent B-cell activation state^5^,^8^. While neutralizing circulating Ig complexes in cirrhotic plasma did not abrogate Fas-sensitization in our experiments with cirrhotic plasma *in vitro*, chronic activation of B-cells by gut-microbial antigens could partially explain the Fas-insensitivity we observed *ex vivo*.

We demonstrate significant crosstalk between TLR pathways and CD40-mediated activation in modulating Fas-mediated apoptosis. B cells do not express membrane-associated CD14(mCD14), but soluble CD14 (sCD14) can directly transfer LPS to myeloid differentiation-2 (MD-2), activating the TLR4 pathway^22^. We have previously shown that competitive inhibition of TLR4-signalling reduces B-cell upregulation of the activation marker HLA-DR^8^. LPS strongly sensitized cirrhotic memory B-cells to apoptosis to a similar degree as CD40 activation, a finding previously demonstrated in murine B-cells^28^ thought to be mediated by increased Fas expression. LPS and LPS-binding protein are well known to be increased in the serum of patients with cirrhosis^23^,^40^, confirmed in this work, and associated with increased rates of infections. We further observed that TLR9-agonism antagonized CD40-mediated Fas-sensitivity, a rescue phenomenon previously shown to be mediated by p38 and Protein Kinase C-dependent pathways that inactivate caspase-8^41^ and upregulate bcl-xl^42^. In other systems, but not examined in this study, IL-4^35^,^43^ and galectin-3^44^ inhibits Fas-mediated apoptosis. BAFF, which is elevated in the serum of cirrhotic patients^45^, in murine B-cells potentiated LPS-induced Fas-sensitivity^28^, but we could not find any evidence that BAFF impacted the survival of HD B-cells exposed to CIR plasma in our experiments.

Contrary to previous experience^24^,^25^,^26^, we found that blocking sFasL in plasma inhibited Fas-mediated apoptosis in healthy donor memory B-cells exposed to cirrhotic plasma suggesting that sFasL can indeed activate Fas in human B-cells. In addition to enhanced expression of mFasL on CD4^+^ T-cells in cirrhosis, sFasL serum levels were markedly elevated and we speculate that sFasL may participate in B-cell apoptosis *in vivo* in cirrhosis. sFasL has been reported to be secreted in exosomes by EBV-transformed B-cells *in vitro*^27^ with cytotoxic effects; however, we found no cytotoxic effect of exosomes separated from cirrhotic plasma except at extreme concentrations and that removal of exosomes enhanced apoptosis suggesting exosomes may harbor anti-apoptotic signals that require further exploration.

There are limitations of this work that we need to acknowledge. Mechanistic studies using human lymphocytes *ex vivo* cannot prove a causal relationship. We did not examine whether acute liver failure could induce similar B-cell abnormalities. Cirrhosis-related apoptosis in other lymphocyte populations was also beyond the scope of the present study.

## Conclusion

The reduction of circulating peripheral CD27^+^ memory B-cell in cirrhosis most likely results from enhanced Fas-mediated apoptosis *in vivo* mediated by increased exposure to cell-bound and surface FasL, and modulated by LPS. Memory B-cell loss and dysfunction in cirrhosis could contribute to cirrhotic immunodeficiency, characterized by increased propensity for bacteremia, sepsis and death.

## Additional Information

**How to cite this article**: Chang, L.-Y. *et al*. Endotoxemia contributes to CD27+ memory B-cell apoptosis via enhanced sensitivity to Fas ligation in patients with Cirrhosis. *Sci. Rep.* **6**, 36862; doi: 10.1038/srep36862 (2016).

**Publisher’s note:** Springer Nature remains neutral with regard to jurisdictional claims in published maps and institutional affiliations.

## Supplementary Material

Supplementary Information

## Figures and Tables

**Table 1:** Demographics and Clinical Characteristics of Study Subjects.

Variable	Healthy donor	Cirrhotic patients	P value
Number of patients	16	26	
Median Age (years, IQR)	54 (49–60)	61 (56–65)	0.05
Ethnicity (W/B/H)	8/8/0	17/9/0	NS
Gender (M/F)	15/1	26/0	NS
Median Alanine Aminotransferase (IU/L) (IQR)	19 (17–25)	46 (30–67)	<0.0001
Median Albumin (g/dl) (IQR)	4.1 (3.8–4.2)	3.8 (3.7–4.1)	NS
Median Total Bilirubin (mg/dl) (IQR)	0.4 (0.5–0.9)	0.7 (0.6–0.9)	NS
Median INR (IQR)	1.1 (1.1–1.1)	1.1 (1.0–1.2)	NS
Median Platelet Count (×10^3^/μl) (IQR)	225 (206–256)	146 (118–177)	<0.0001
Etiology (Viral/Metabolic)	—	16/10	

NS: not significant.
