# Supplementary material for: Endotoxemia contributes to CD27+ memory B-cell apoptosis via enhanced sensitivity to Fas ligation in patients with Cirrhosis
Source: Sci Rep. 2016 Nov 18;6:36862. doi: 10.1038/srep36862 (PMC5114671; doi:10.1038/srep36862)
Supplement: Supplementary Information [file srep36862-s1.pdf]

Title: Endotoxemia contributes to CD27+ memory B-cell apoptosis via enhanced sensitivity to Fas ligation in patients with Cirrhosis

Authors:

Li-Yuan Chang[1,2], Yonghai Li[1,2], David E. Kaplan[1,2*]

[1]Medicine and Research Services, Corporal Michael J. Crescenz VA Medical Center, 3900 Woodland Avenue, Philadelphia, PA 19104

[2]Division of Gastroenterology, Department of Medicine, University of Pennsylvania, 9th floor BRB, 421 Curie Blvd. Philadelphia, PA 19104

*** Corresponding Author:** David E. Kaplan, MD MSc, Philadelphia VA Medical Center, Research Bldg 21, A402A, 3900 Woodland Avenue, Philadelphia, PA 19104, +1-215-823-5800 6729, dakaplan@mail.med.upenn.edu

SUPPLEMENTARY MATERIAL

Supplemental Figure 1

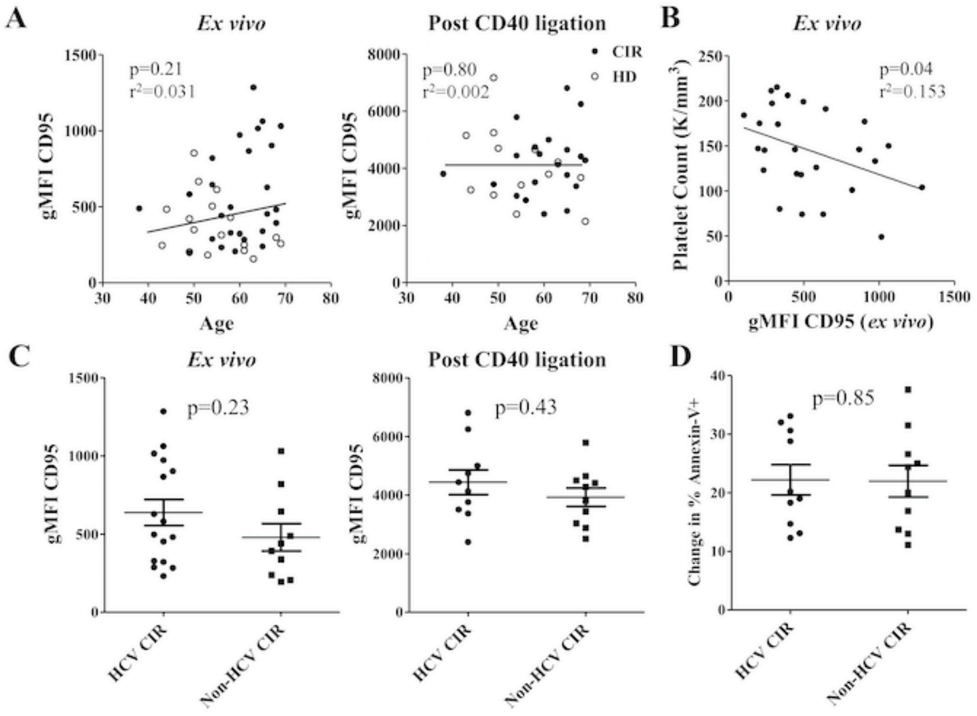

Supplemental Figure 1. Age and cirrhosis etiology do not affect basal or activation induced CD95 expression. (A) Spearman correlation of patient age and gMFI of CD95 in healthy donors (open circles) and cirrhotic patients (filled circles) *ex vivo* (left) and after CD40-mediated activation (right). (B) Spearman correlation of platelet count (K/mm^3) and gMFI of CD95 *ex vivo*. (C) Comparison of gMFI of CD95 in hepatitis C- and non-viral-related cirrhosis *ex vivo* (left) and after CD40-mediated activation (center). p-value by Wilcoxon test. (D) Comparison of change in %Annexin-V$^+$ memory B-cells between viral- and non-viral cirrhosis after CD40-mediated activation (right). P-value by Wilcoxon test.

Supplemental Figure 2

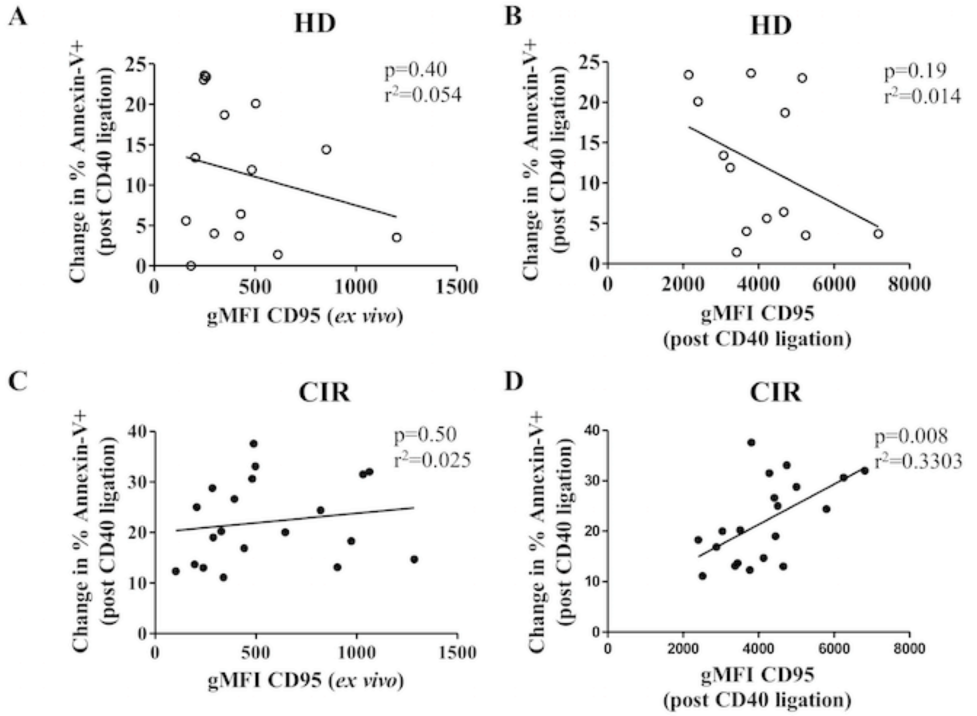

Supplemental Figure 2. Sensitivity to Fas-mediated apoptosis correlates with post-activation rather than basal CD95 expression. Spearman correlation of %Annexin-V^+ memory B-cells after Fas-exposure with (A) *ex vivo* Fas expression (gMFI of CD95) in healthy donors (HD), (B) post-CD40-activation Fas expression in healthy donors, (C) *ex vivo* Fas expression (gMFI of CD95) in cirrhotic patents (CIR), and (D) post-CD40-activation Fas expression in cirrhotic patients.

Supplemental Figure 3

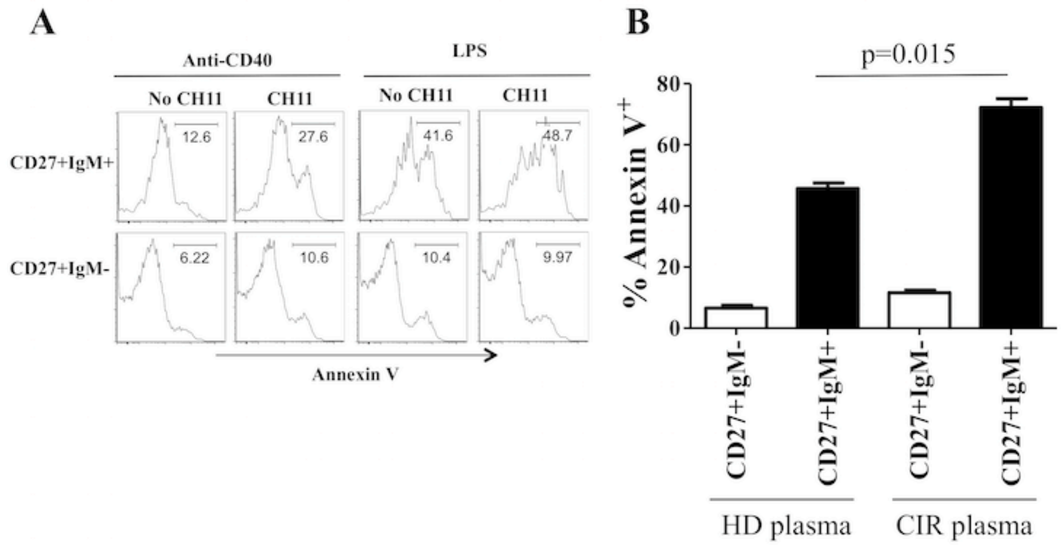

Supplemental Figure 3. Sensitivity to Fas-mediated apoptosis among $CD27^{+}IgM^{+}$ and $CD27^{+}IgM^{-}$ B-cells. (A) CIR $CD27^{+}$ B-cells were stimulated for 48h with either agonist anti-CD40 or LPS then incubated with anti-Fas (CH11) or control for 18h. Representative histograms showing Annexin-V expression in IgM^{+} and IgM^{-} subsets are shown. Representative data from 3 cirrhotic patients tested shown. (B) HD $CD27^{+}$ were isolated and incubated for 18h with either HD or CIR plasma. The percentage of $CD27^{+}IgM^{+}$ and $CD27^{+}IgM^{-}$ Annexin-V+ cells are shown. P-value for difference between HD and CIR by Wilcoxon test.

Supplemental Figure 4

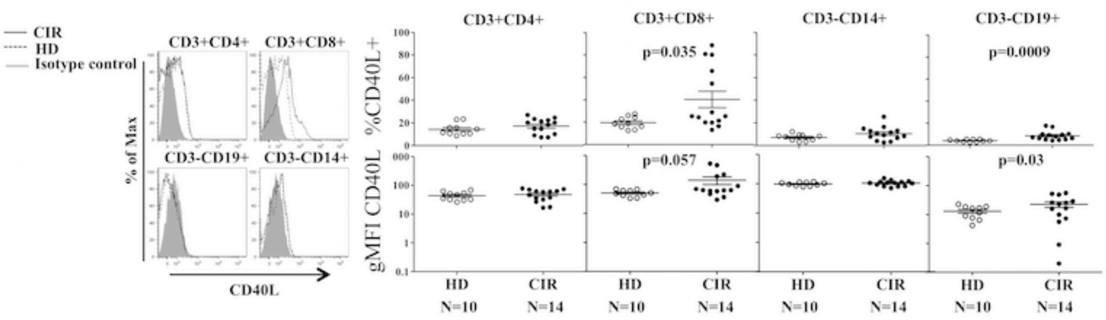

Supplemental Figure 4. mCD40L expression on PBMC subsets. Representative histograms and quantification of CD40L expression on $CD3^{+}CD4^{+}$ cells, $CD3^{+}CD8^{+}$ cells, $CD3^{-}CD14^{+}$ cells and $CD3^{-}CD19^{+}$ cells in HD and CIR.
